# Supplementary material for: Performance of arsenene and antimonene double-gate MOSFETs from first principles
Source: Nat Commun. 2016 Aug 25;7:12585. doi: 10.1038/ncomms12585 (PMC5007351; doi:10.1038/ncomms12585)
Supplement: Supplementary Information — Supplementary Figures 1-7, Supplementary Tables 1-4, Supplementary Notes 1-10 and Supplementary References [file ncomms12585-s1.pdf]

Supplementary Figures

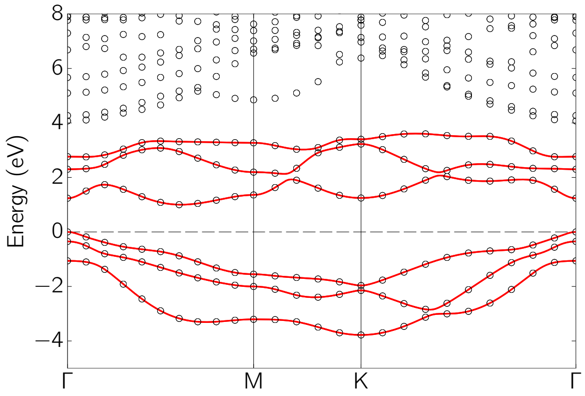

Supplementary Figure 1: Energy bands of antimonene along a high-symmetry path in the Brillouin zone, including spin-orbit coupling effects. Empty circles denote the results of a direct DFT calculation while red solid lines represent the Wannier-interpolated bands. The zero of energy is set to the top of the valence bands.

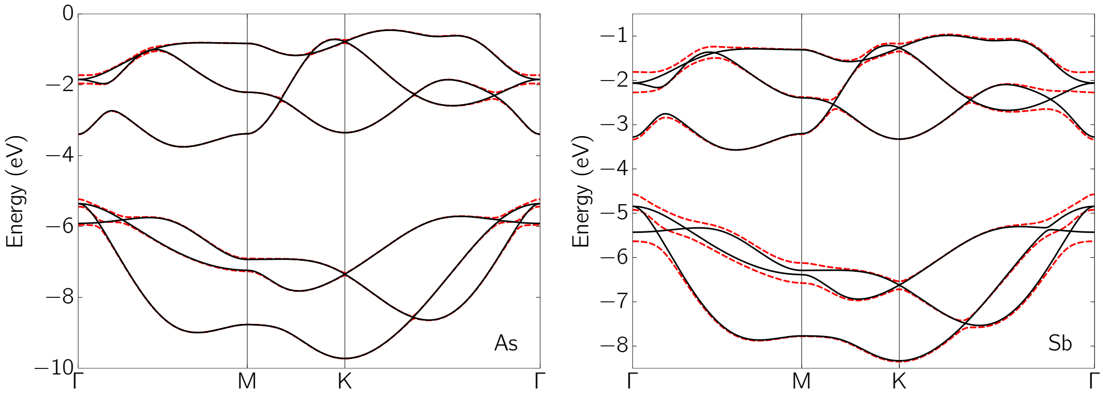

Supplementary Figure 2: Wannier-interpolated energy bands of arsenene (left panel) and antimonene (right panel) along a high-symmetry path in the Brillouin zone, comparing the band structure calculated including (red dashed lines) and disregarding (solid black lines) spin-orbit coupling effects. The bandgaps without SOC are 1.62 eV and 1.29 eV for As and Sb, respectively, while the valence-band splitting due to the SOC is 0.208 eV and 0.349 eV, respectively. The values of the bandgaps with SOC are reported in the main text. The zero of energy is set at the vacuum level.

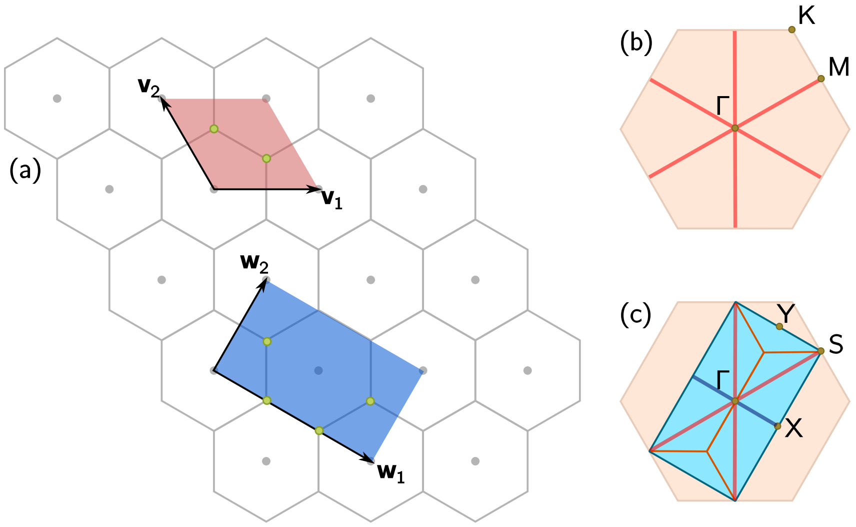

Supplementary Figure 3: (a) Primitive unit cell with two atoms (top), and rectangular supercell with four atoms (bottom) used when applying strain in a given direction β. In particular, $\mathbf{w}_1$ ($\mathbf{w}_2$) is along an armchair (zigzag) direction. Both $\mathbf{v}_1$ and $\mathbf{v}_2$ are along zigzag directions, instead. (b) Brillouin zone of the primitive unit cell. The six equivalent $\Gamma-$M lines are highlighted in red. (c) Folding of the Brillouin zone when the 4-atom supercell is considered (light blue rotated rectangle), and labelling of the high-symmetry points. The six $\Gamma-$M lines of panel (b) split into four $\Gamma-$S lines (red) and two $\Gamma-$X lines (dark blue).

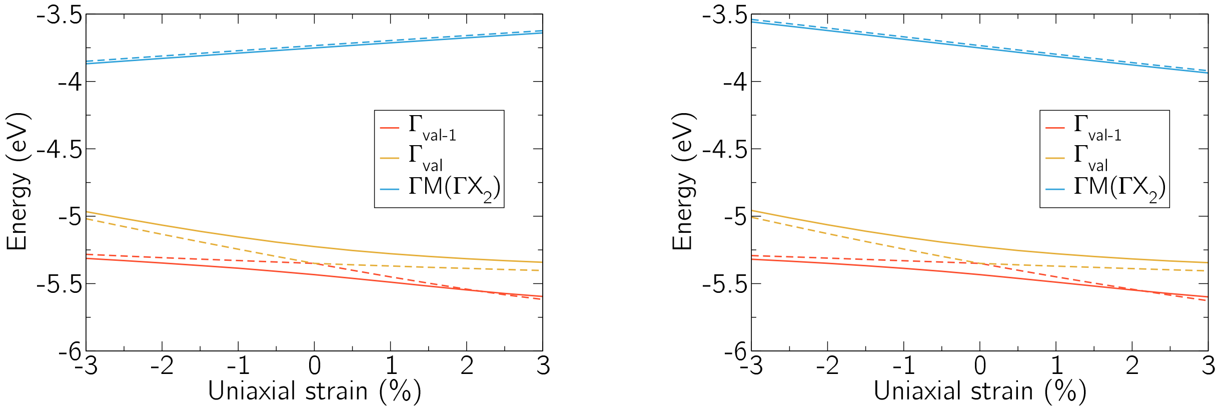

Supplementary Figure 4: Vacuum-level-corrected band edges as a function of the strain for the As monolayers. Left panel: strain along the $\mathbf{w}_1$ direction (armchair). Right panel: strain along the $\mathbf{w}_2$ direction (zigzag). Γ_{val} indicates the topmost valence band at Γ, $\Gamma_{val\text{-}1}$ indicates the second valence band. Dashed curves refer to the case without SOC, while solid curves include SOC effects.

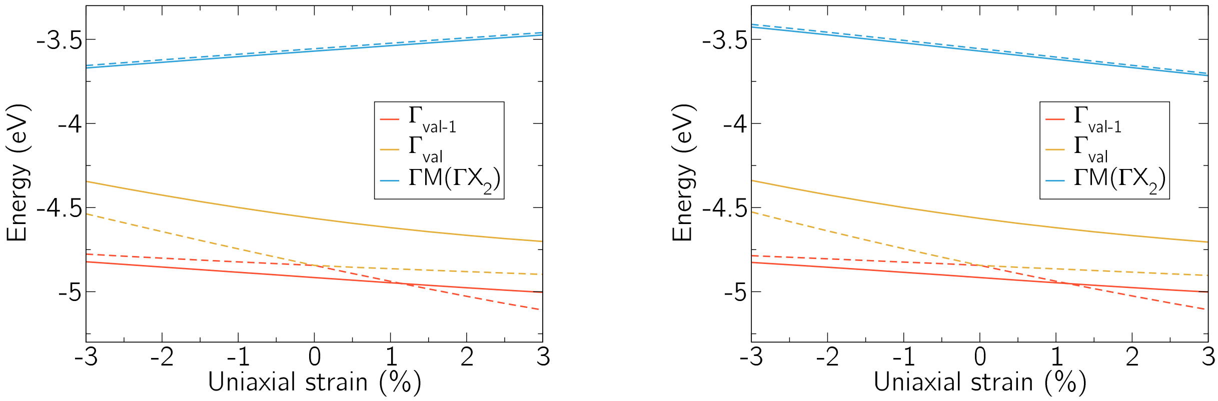

Supplementary Figure 5: Vacuum-level-corrected band edges as a function of the strain for the Sb monolayers. Left panel: strain along the $\mathbf{w}_1$ direction (armchair). Right panel: strain along the $\mathbf{w}_2$ direction (zigzag). Γ_{val} indicates the topmost valence band at Γ, $\Gamma_{\text{val-1}}$ indicates the second valence band. Dashed curves refer to the case without SOC, while solid curves include SOC effects.

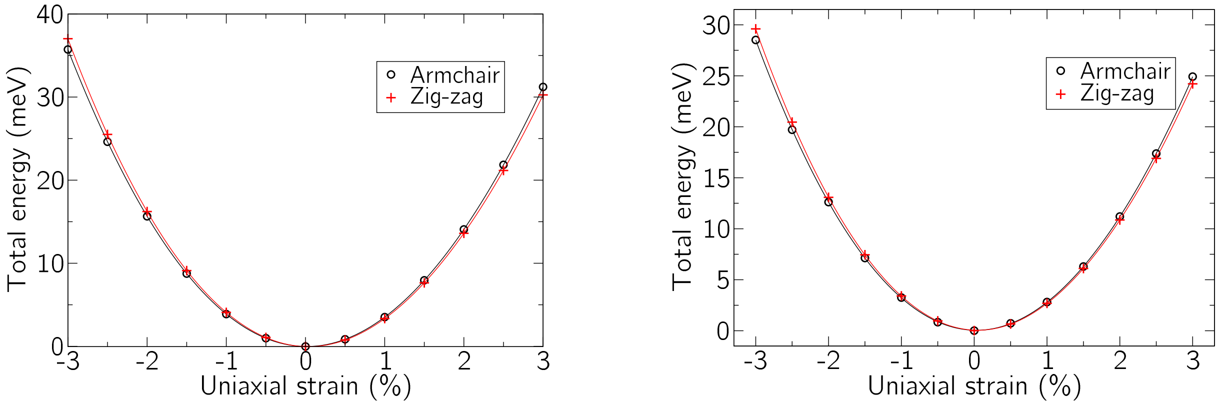

Supplementary Figure 6: Total energy as a function of the uniaxial strain (when SOC effects are not included) in the armchair (open points) and zigzag (plus symbols) direction. Left panel: As monolayer; right panel: Sb monolayer. The curves are the cubic fits of the data points used to obtain the elastic moduli.

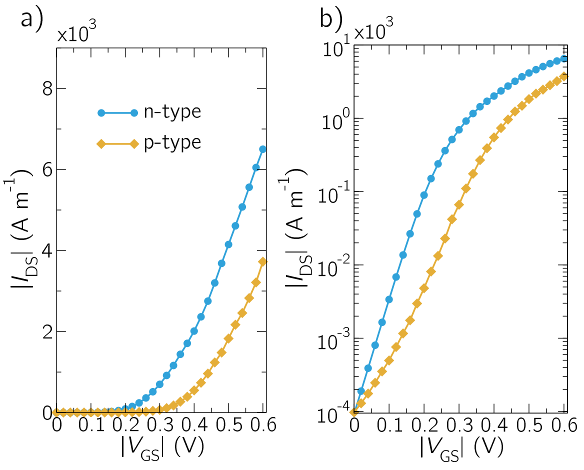

Supplementary Figure 7: $|I_{\text{DS}}| - |V_{\text{GS}}|$ curve in (a) linear and (b) semi-logarithmic scale for As n-MOS and p-MOS transistors and a gate length of L_{G}=7 nm. Sb FETs show a similar behaviour and are not shown here.

Supplementary Tables

Supplementary Table 1: Deformation potentials (in eV) for the valence and conduction bands of As and Sb monolayers, both including and disregarding spin-orbit coupling effects. For valence bands with spin-orbit coupling, we only report the deformation potential of the topmost valence band; in the case without spin-orbit coupling, $i = 1, 2$ are defined so that $i = 1$ is the topmost valence band for positive strain, and $i = 2$ the second highest valence band for positive strain, equivalent to saying that we chose $i = 1, 2$ so that $E_\beta^{\rm val}(i = 2) < E_\beta^{\rm val}(i = 1)$. Results without spin-orbit coupling are consistent with previous calculations at the same level of theory [1].

With SOC	$E_\beta^{\rm val}$	$E_\beta^{\rm cond}(\Gamma - {\rm X})$
As (β = armchair)	-6.243	3.815
As (β = zigzag)	-6.386	-6.351
Sb (β = armchair)	-5.966	3.265
Sb (β = zigzag)	-6.078	-4.843

Without SOC	$E_\beta^{\rm val}(i = 1)$	$E_\beta^{\rm val}(i = 2)$	$E_\beta^{\rm cond}(\Gamma - {\rm X})$
As (β = armchair)	-1.997	-10.094	3.785
As (β = zigzag)	-1.922	-10.321	-6.380
Sb (β = armchair)	-2.006	-9.571	3.260
Sb (β = zigzag)	-1.969	-9.674	-4.895

Supplementary Table 2: 2D elastic moduli of the As and Sb monolayer systems, both including and disregarding spin-orbit coupling effects.

	S_0 (Å^2)	C_β (eV Å^{-2}) with SOC	C_β (eV Å^{-2}) without SOC
As (β = armchair)	22.5	3.271	3.299
As (β = zigzag)		3.279	3.308
Sb (β = armchair)	29.4	1.948	2.012
Sb (β = zigzag)		1.945	2.019

Supplementary Table 3: Effective masses of the relevant bands of arsenene and antimonene, in units of the electron mass m_0, when SOC is not included. Symbols are explained in the text. Note that $m_{\rm DOS}^{\rm c}$ is the effective DOS mass for each of the 6 identical conduction band valleys, while $m_{\rm DOS}^{\rm v}$ represents the total effective DOS mass for the two degenerate valence bands.

	As	Sb
$m_{\rm DOS}^{\rm c}$	0.273	0.260
$m_{\rm L}^{\rm c}$	0.508	0.461
$m_{\rm T}^{\rm c}$	0.150	0.149
$m_{\rm DOS}^{\rm v}$	0.554	0.523
$m_{\rm HH}^{\rm v}$	0.482	0.443
$m_{\rm LH}^{\rm v}$	0.077	0.073

Supplementary Table 4: Mobilities for arsenene and antimonene, estimated at $T = 300\,{\rm K}$, in units of cm^{2}V^{-1}s^{-1}. Both the values obtained with and without spin-orbit coupling (SOC) are reported.

	With SOC		Without SOC	
	$\mu^{\rm val}$	$\mu^{\rm cond}$	$\mu^{\rm val}$	$\mu^{\rm cond}$
As	1700	635	1355	622
Sb	1737	630	946	641

Supplementary Note 1: Band structure of antimonene, and spin-orbit coupling effects

In the paper, only the band structure of arsenene has been shown, because the band structure of antimonene is qualitatively the same. For completeness we report in this Supplementary Information, in Supplementary Figure 1, the band structure of antimonene as well as the bands interpolated using maximally-localised Wannier functions. Moreover, we compare all results with the case in which the spin-orbit coupling (SOC) has been set to zero, showing that the SOC almost does not change the final results for the mobilities in the conduction band, while it significantly affects results in the valence (due to the splitting of the degeneracies). In particular, in Supplementary Figure 2 we compare the (Wannier-interpolated) band structures of As and Sb, to show the effect of the spin-orbit coupling on the electronic bands.

Supplementary Note 2: Calculation of mobilities

We describe in detail in this Section how we calculated the phonon-limited mobilities of the 2D systems considered in the paper.

We calculate mobilities using the Boltzmann transport equation, where the relaxation time for scattering with LA phonons is computed using deformation potential theory [2] in the effective mass approximation. For a single-valley, non-degenerate band, the reciprocal of the scattering time with LA phonons propagating in the β direction for the $i-$th band at $\mathbf{k}-$point $\mathbf{k}$ is given by [2, 3]:

$$\frac{1}{\tau_\beta(i,\mathbf{k})} = \frac{2\pi k_\mathrm{B} T (E^i_\beta)^2}{\hbar C_\beta} \sum_{\mathbf{k}'} \delta\left[\varepsilon_i(\mathbf{k}) - \varepsilon_i(\mathbf{k}')\right](1-\cos\theta), \tag{1}$$

where k_B is the Boltzmann constant, E^i_β is the deformation potential of the $i-$th band for deformations in the β direction, C_β is the 2D elastic modulus for strains along β, $\varepsilon_i(\mathbf{k})$ is the energy of the $i-$th band at $\mathbf{k}$, and we are replacing for simplicity the scattering angle weighting factor with $(1-\cos\theta)$ (valid for a spherical energy surface, where θ is the angle between $\mathbf{k}$ and $\mathbf{k}'$).

The calculation of the various coefficients is described in the next sections. To get the value of the mobility, though, it is easier to work out the formula in the specific case of a 2D system. By replacing the sum over $\mathbf{k}'$ with an integral, and then passing from an integral over the Brillouin Zone to an integral over energies, we obtain that

$$\frac{1}{\tau_\beta} = \frac{k_\mathrm{B} T (E^i_\beta)^2 (m^*_\mathrm{DOS})_i}{\hbar^3 C_\beta}. \tag{2}$$

Finally, the mobility μ is diagonal relative to the axes of the effective mass of the valley of interest [4], and its diagonal components can be obtained as

$$\mu_{\beta\beta} = e\,\langle\tau_\beta\rangle \cdot \left(\frac{1}{m^*}\right)_{\beta\beta} \tag{3}$$

with e being the electron charge, $(1/m^*)$ the inverse effective mass tensor, and $\langle\tau\rangle$ the average scattering time (as defined in Eq. (16) of Ref. [4]). The formula is valid in the same reference frame in which the effective mass tensor is diagonal. Note that in the 2D case, $\langle\tau\rangle = \tau$ since there is no energy dependence.

In the case of multiple valleys or degenerate bands, the expression for the mobility is the same of Supplementary Equation (3), but applies to a single valley. To understand how the mobilities need to be added, it is easier to write them in terms of the electrical conductivity σ, using the fact that the total conductivity is the sum of the conductivities of the different channels. To do so, we notice that we can write the mobility μ^i of Supplementary Equation (3) as

$$\mu^i = \frac{\sigma^i}{n^i e}, \tag{4}$$

for each valley i contributing to the transport, where n^i is the charge in the $i-$th valley. Using $\sigma_\mathrm{T} = \sum_i \sigma^i$, where σ_T represents the total conductivity, we obtain therefore for the total mobility:

$$\mu_\mathrm{T} = \frac{\sum_i \mu^i n^i}{\sum_i n^i}. \tag{5}$$

Supplementary Note 3: Relevant band edges: generic discussion

As already discussed in the paper, the bands of As and Sb are quite similar, so the following discussion applies to both systems, unless explicitly mentioned.

As it is visible from the band structure of the two materials (see Supplementary Figure 1, and Figure 1 in the main paper), the relevant band edge to consider in the conduction is the minimum along the $\Gamma-$M line. All other conduction band edges (for instance, the minimum at Γ or at the K point) are several hundreds of meV above the one along $\Gamma-$M, and therefore do not contribute to transport at room temperature for typical doping levels. We have also verified that this condition holds at least for a strain range between -1 and 1%. In the unstrained case, this minimum is composed of six degenerate valleys, along the six equivalent $\Gamma-$M lines. In the rectangular supercell shown in blue in Supplementary Figure 3(a), two of these lines fold along the $\Gamma-$X line, while four fold on the $\Gamma-$S lines, as shown in Supplementary Figure 3. When a uniaxial strain is applied, the degeneracy is lost and the six valleys split in two groups (the two $\Gamma-$X and the four $\Gamma-$S valleys), with different deformation potentials. We also stress that in the rectangular cell, the $\Gamma-$S line is not anymore a high-symmetry line, so the band edge can also move out of this line. Note that, actually, for the evaluation of the mobility we just need the deformation potential along the two principal axes of the effective mass, that is, we just need the values for the $\Gamma-$X lines, both for strains along the armchair and zigzag directions. This discussion holds both with and without SOC.

In the valence, in the case without SOC, the only relevant band edges are the two degenerate maxima at Γ (also in this case other local maxima are further down in energy and can be disregarded for the calculation of the mobility). These are both single-valley maxima, but they split when a uniaxial strain is applied because they have different deformation potentials. When including SOC effects, the degeneracy is split and, for realistic band filling levels ($\lesssim 10^{13}$ cm^{-2}), we can limit our calculations only to the topmost valence band, since it is the only ones going to be filled.

Let us now compute an explicit formula for the mobilities for the As and Sb monolayer systems:

Supplementary Note 4: Relevant conduction band edges

The mass tensor of each of the 6 $\Gamma-$M valleys is non-isotropic, with a longitudinal mass $m_{\mathrm{L}}^{\mathrm{c}}$ along the $\Gamma-$M direction, and a transverse mass $m_{\mathrm{T}}^{\mathrm{c}}$ in the orthogonal direction. The inverse-mass tensor in a basis set where the first vector is along the longitudinal direction is therefore:

$$\left(\frac{1}{m^*}\right)_{ij} = \begin{pmatrix} \frac{1}{m_{\mathrm{L}}^{\mathrm{c}}} & 0 \\ 0 & \frac{1}{m_{\mathrm{T}}^{\mathrm{c}}} \end{pmatrix}. \tag{6}$$

In this basis set, the two τ_β to calculate are along the longitudinal direction (corresponding to strains along the armchair direction, see Supplementary Figure 3), and along the transverse direction (zigzag). Therefore, substituting Supplementary Equation (2) into Supplementary Equation (3), the mobility tensor for a single valley, in this basis set, will be:

$$\boxed{\mu^{\mathrm{cond},i} = \frac{e\hbar^3}{k_{\mathrm{B}} T m_{\mathrm{DOS}}^{\mathrm{c}}} \begin{pmatrix} \frac{C_{\mathrm{armchair}}}{(E_{\mathrm{armchair}}^{\mathrm{cond}})^2 m_{\mathrm{L}}^{\mathrm{c}}} & 0 \\ 0 & \frac{C_{\mathrm{zigzag}}}{(E_{\mathrm{zigzag}}^{\mathrm{cond}})^2 m_{\mathrm{T}}^{\mathrm{c}}} \end{pmatrix}}, \tag{7}$$

with $m_{\mathrm{DOS}}^{\mathrm{c}}$ being the DOS mass of a single conduction valley.

Since the DOS effective mass is the same for each valley and the valleys are degenerate (in the absence of strain that splits the bands, as discussed above), all valleys have the same population $n^i = n/6$ and therefore the total conductivity is simply:

$$\mu_{\mathrm{T}}^{\mathrm{cond}} = \frac{1}{6}\sum_{i=0}^{5} \mu^{\mathrm{cond},i}, \tag{8}$$

where the tensors $\mu^{\mathrm{cond},i}$ must, however, be rotated. We remind here that given a tensor $\begin{pmatrix} \alpha & 0 \\ 0 & \beta \end{pmatrix}$ in a reference frame in which it is diagonal, its form in a frame rotated counterclockwise by an angle θ is

$$\begin{pmatrix} \alpha\cos^2\theta + \beta\sin^2\theta & (\beta-\alpha)\cos\theta\sin\theta \\ (\beta-\alpha)\cos\theta\sin\theta & \alpha\sin^2\theta + \beta\cos^2\theta \end{pmatrix}. \tag{9}$$

Choosing the reference frame in which $\mu^{\text{cond},0}$ is diagonal, we have to sum the 6 bands, each rotated by $\frac{\pi}{3}$ with respect to the previous one, and therefore

$$(\mu_{\mathrm{T}}^{\text{cond}})_{11} = \frac{\alpha}{6}\sum_{i=0}^{5}\cos^2\left(i\cdot\frac{\pi}{3}\right) + \frac{\beta}{6}\sum_{i=0}^{5}\sin^2\left(i\cdot\frac{\pi}{3}\right). \tag{10}$$

Now $\sum_{i=0}^{5}\sin^2\left(i\cdot\frac{\pi}{3}\right) = 0+\frac{3}{4}+\frac{3}{4}+0+\frac{3}{4}+\frac{3}{4} = 3$ and $\sum_{i=0}^{5}\cos^2\left(i\cdot\frac{\pi}{3}\right) = \sum_{i=0}^{5} 1-\sin^2\left(i\cdot\frac{\pi}{3}\right) = 6-3 = 3$, and therefore $(\mu_{\mathrm{T}}^{\text{cond}})_{11} = (\mu_{\mathrm{T}}^{\text{cond}})_{22} = \frac{\alpha+\beta}{2}$. Similarly, one can show that the off-diagonal contributions cancel in pairs, and therefore the total conduction mobility is a multiple of the identity, with value

$$\boxed{\mu_{\mathrm{T}}^{\text{cond}} = \frac{e\hbar^3}{2k_{\mathrm{B}}Tm_{\mathrm{DOS}}^{\mathrm{c}}}\left(\frac{C_{\text{armchair}}}{(E_{\text{armchair}}^{\text{cond}})^2 m_{\mathrm{L}}^{\mathrm{c}}} + \frac{C_{\text{zigzag}}}{(E_{\text{zigzag}}^{\text{cond}})^2 m_{\mathrm{T}}^{\mathrm{c}}}\right).} \tag{11}$$

The fact that the μ_{T} tensor is isotropic is expected, because the system has hexagonal symmetry. Note that averaging on all valleys is equivalent to averaging the tensor of a single valley in all directions, as it is the case also for cubic systems [4].

Supplementary Note 5: Relevant valence band edges

Without SOC, in the valence band we have instead two single valleys that are degenerate at Γ, but with different masses (and scattering times), that we can call light holes (LH) and heavy holes (HH). We assume that both bands are parabolic, and in this case the 2D density of states is a step function (where the step height, occurring at the band edge energy, is proportional to the 2D DOS effective mass). Then, the population n_i of a given band is simply

$$n_i = D\cdot m_{\mathrm{DOS}}^{i} \tag{12}$$

where D is a constant that contains the energy difference between the chemical potential and the band edge. (We are assuming that this energy difference is the same for both bands, true if intraband scattering events can quickly equilibrate the bands so that they have the same chemical potential, and if we consider an unstrained system so that the two LH and HH bands are degenerate at their maximum).

Replacing this simple expression for n_i in Supplementary Equations (3) and (5), the mobility is:

$$\mu_\beta^{\text{val}} = e\cdot\frac{\frac{Dm_{\mathrm{DOS}}^{\mathrm{LH}}\tau_\beta^{\mathrm{LH}}}{(m_{\mathrm{LH}}^*)_\beta} + \frac{Dm_{\mathrm{DOS}}^{\mathrm{HH}}\tau_\beta^{\mathrm{HH}}}{(m_{\mathrm{HH}}^*)_\beta}}{D(m_{\mathrm{DOS}}^{\mathrm{LH}} + m_{\mathrm{DOS}}^{\mathrm{HH}})} = e\cdot\frac{\frac{m_{\mathrm{DOS}}^{\mathrm{LH}}\tau_\beta^{\mathrm{LH}}}{(m_{\mathrm{LH}}^*)_\beta} + \frac{m_{\mathrm{DOS}}^{\mathrm{HH}}\tau_\beta^{\mathrm{HH}}}{(m_{\mathrm{HH}}^*)_\beta}}{m_{\mathrm{DOS}}^{\mathrm{v}}} \tag{13}$$

where $m_{\mathrm{DOS}}^{\mathrm{v}}$ is the total DOS mass in the valence. Finally, we can prove that in the valence the mobility does not depend on the DOS mass of each of the two valleys, but only on the total DOS mass and on the effective masses in the transport direction. In fact, by simply replacing Supplementary Equation (2), one finally obtains:

$$\boxed{\mu_\beta^{\text{val}}[\text{without SOC}] = \frac{e\hbar^3 C_\beta}{k_{\mathrm{B}}T}\cdot\frac{\frac{1}{(E_\beta^{\mathrm{LH}})^2(m_{\mathrm{LH}}^*)_\beta} + \frac{1}{(E_\beta^{\mathrm{HH}})^2(m_{\mathrm{HH}}^*)_\beta}}{m_{\mathrm{DOS}}^{\mathrm{v}}}}. \tag{14}$$

When we include spin-orbit effects, we need to consider only the topmost valence band (as already discussed), and since the band is isotropic, we can also set its mass $m_{\mathrm{v}}^* = m_{\mathrm{DOS}}^{\mathrm{v}}$. The formula then simplifies to:

$$\boxed{\mu_\beta^{\text{val}}[\text{with SOC}] = \frac{e\hbar^3 C_\beta}{k_{\mathrm{B}}T}\cdot\frac{1}{(E_\beta^{\mathrm{LH}})^2(m_{\mathrm{v}}^*)^2}}. \tag{15}$$

Supplementary Note 6: Calculation of the deformation potential

The deformation potential is calculated using finite differences starting from band energies calculated with DFT of systems strained along the two relevant transport directions (zigzag and armchair). Considering a given band edge i (for instance the topmost valence band at Γ, or the conduction band along the Γ−M

line in As and Sb), we can define $\Delta V_\beta^i = \varepsilon^i(\Delta l_\beta) - \varepsilon^i(\Delta l_\beta = 0)$, where $\varepsilon^i(\Delta l_\beta)$ is the energy of the $i-$th band edge for a system strained in the β direction by a quantity Δl_β. The deformation potential is then simply $E_\beta^i = \Delta V_\beta^i/(\Delta l_\beta/l_{\beta,0})$, where $l_{\beta,0}$ is the relaxed value of the lattice constant in the β direction, in the limit of small strains Δl_β. It is important to stress that the band-edge energies directly extracted from DFT calculations of different systems are ill-defined, because the position of the vacuum level can change in each calculation. We therefore use always vacuum-level-corrected band energies ε^i, obtained by defining, at each strain β, the vacuum level as the zero of energy. The vacuum level is obtained by calculating the averaged electrostatic potential in the region of space far away from the 2D layers. We verified that in all cases such potential is flat (within a 0.01 meV precision) $4-5$ Å away from the monolayers. We also note that the primitive 2D cell (with 2 atoms per cell) has non-orthogonal lattice vectors. In order to define strained systems in the two transport directions, we define a rectangular cell with 4 atoms per cell, where the two lattice vectors $\mathbf{w}_1$ and $\mathbf{w}_2$ are in the armchair and zigzag direction respectively, as shown in Supplementary Figure 3. Uniaxial strains are then applied to this rectangular supercell.

We consider 13 calculations for different, uniformly spaced strains between -3% and 3% for each of the two β directions, and we extract the deformation potential from the linear coefficient of a quadratic fit of the vacuum-level-corrected band edges, as shown in Supplementary Figs. 4 and 5, where we report results both with and without SOC.

Supplementary Note 7: Effective masses

We have computed the effective masses at zero strain in the primitive unit cell; we have checked that the effective masses do not change significantly with strain. The values (calculated as described below) are reported in Table 1 of the main paper when including SOC effects. We also report here, in Supplementary Table 3, the values calculated when SOC is not included.

Also in the case of effective masses, we have to distinguish two cases. In the conduction band, the masses can be calculated by a parabolic fit of the band energies along the two directions. We have used for the fit 21 $\mathbf{k}-$points around the band minimum (at zero strain) in the longitudinal and transverse directions. The DOS mass $m_{\mathrm{DOS}}^{\mathrm{c}}$ is instead obtained by a parabolic fit of the integrated DOS (to take into account non-parabolicity effects). The DOS has been calculated on a dense $\mathbf{k}-$mesh using Wannier interpolation. The conduction valleys are in a very good approximation parabolic and indeed $m_{\mathrm{DOS}}^{\mathrm{c}} \approx \sqrt{m_{\mathrm{L}}^{\mathrm{c}} m_{\mathrm{T}}^{\mathrm{c}}}$ (see Table 1 in the main paper).

In the valence band, without SOC and in the absence of strain we have two isotropic bands with two different masses, named heavy hole (HH) and light hole (LH) for the larger and smaller mass (in absolute value), respectively. A difficulty arises when we apply some strain: in this case, the two bands split and we need to know how to associate the lower-energy and higher-energy valence bands to corresponding effective mass (LH or HH). Actually, with strain the two masses become anisotropic tensors. In particular, given a direction β for the strain, we have checked that (both for As and Sb) the effective mass obtained fitting the bands for $\mathbf{k}-$points in the same direction as β is HH for the bands with larger deformation potential (smaller in absolute value, since $E < 0$), while LH for the band with smaller deformation potential. Instead, if the $\mathbf{k}-$points are taken in a direction orthogonal to β, the two masses HH and LH are reversed. Since in Supplementary Equation (14) we need only the deformation potential for strains along the same transport direction as the effective mass components, Supplementary Equation (14) becomes:

$$\mu_\beta^{\mathrm{val}} = \frac{e\hbar^3 C_\beta}{k_{\mathrm{B}} T} \cdot \frac{\frac{1}{(E_\beta(v,i=1))^2 (m_{\mathrm{HH}}^*)_\beta} + \frac{1}{(E_\beta(\mathrm{v},i=2))^2 (m_{\mathrm{LH}}^*)_\beta}}{m_{\mathrm{DOS}}^{\mathrm{v}}}, \tag{16}$$

where we have defined $i = 1, 2$ consistently to Supplementary Table 1, i.e., so that $E_\beta(\mathrm{v}, i = 2) < E_\beta(\mathrm{v}, i = 1)$.

In the case with SOC, this difficulty does not arise because the two bands do not cross.

Supplementary Note 8: Elastic moduli

The 2D elastic modulus in a given direction β is defined as:

$$C_\beta = \frac{1}{S_0} \left. \frac{\partial^2 \mathcal{E}_{\Delta l_\beta}}{\partial(\Delta l_\beta/l_{\beta,0})^2} \right|_{\Delta l_\beta = 0}, \tag{17}$$

where $\mathcal{E}_{\Delta l_\beta}$ is the total energy of a system strained uniaxially by $\Delta l_\beta / l_{\beta,0}$ in the β direction, and S_0 is the value of the unstrained unit-cell surface.

The values reported in the paper are obtained from the parabolic coefficient of a cubic fit of the total energy as a function of strain in the $[-3, +3]\%$ range for the four-atom supercells, shown in Supplementary Figure 6 for both materials (when SOC is included). In a hexagonal system the elastic tensor should be isotropic in the plane. Indeed, apart from numerical inaccuracies, the C_β values are the same for both directions (see Supplementary Table 2), and do not significantly change including or disregarding SOC.

Supplementary Note 9: Values of the mobility

Using the values of Supplementary Table 1, Table 1 (in the main paper) and Supplementary Table 2, calculated as described in the previous sections, we estimated the values for the mobilities at $T = 300\,\mathrm{K}$, in units of $\mathrm{cm^2 V^{-1} s^{-1}}$, that we report in Supplementary Table 4. Note that since we expect that the total mobility is isotropic, we indicate only the average value obtained for the armchair and the zigzag directions. In any case, the values in the two directions differ (due to numerical errors) by less than 5%.

For completeness, we also report the value of the single-valley conduction mobility tensor, including spin-orbit coupling effects, in a basis set where the first vector is along the armchair direction and the second along the zigzag one, and expressed in units of $\mathrm{cm^2 V^{-1} s^{-1}}$:

$$\mu_{\mathrm{As}}^{\mathrm{cond},i} = \begin{pmatrix} 567 & 0 \\ 0 & 704 \end{pmatrix}, \qquad \mu_{\mathrm{Sb}}^{\mathrm{cond},i} = \begin{pmatrix} 507 & 0 \\ 0 & 753 \end{pmatrix}. \tag{18}$$

Using the formulas above, one can also calculate the scattering times of electrons with LA phonons, that turn out to be (in conduction): $\tau^{\mathrm{As}}_{\mathrm{armchair}} = 161\,\mathrm{fs}$, $\tau^{\mathrm{As}}_{\mathrm{zigzag}} = 58\,\mathrm{fs}$, $\tau^{\mathrm{Sb}}_{\mathrm{armchair}} = 136\,\mathrm{fs}$, $\tau^{\mathrm{Sb}}_{\mathrm{zigzag}} = 62\,\mathrm{fs}$. Finally, in order to estimate the mean free path, it is useful to evaluate the Fermi velocity for electrons in the $\Gamma-\mathrm{M}$ valleys, that for electrons along the i principal direction of the valley is given by:

$$v_i^{\mathrm{F}} = \sqrt{\frac{2\pi\hbar^2 n_{\mathrm{dop}}}{m_i m_{\mathrm{DOS}}}}, \tag{19}$$

where we have used the 2D DOS to relate the the effective doping of the system n_{dop} to the Fermi energy, and where m_i indicates the effective mass in the i direction. As a reference value, for a doping of $n_{\mathrm{DOP}} = 5 \cdot 10^{13}\,\mathrm{cm^{-2}}$, we obtain for As: $v_{\mathrm{L}}^{\mathrm{F}} = 0.55\,\mathrm{nm\,fs^{-1}}$, $v_{\mathrm{T}}^{\mathrm{F}} = 1.01\,\mathrm{nm\,fs^{-1}}$, while for Sb: $v_{\mathrm{L}}^{\mathrm{F}} = 0.60\,\mathrm{nm\,fs^{-1}}$, $v_{\mathrm{T}}^{\mathrm{F}} = 1.04\,\mathrm{nm\,fs^{-1}}$.

Supplementary Note 10: n-MOS and p-MOS Field Effect Transistors

In order to span the whole device parameter space and optimise the device performances, we compare the $I - V$ curves of n-MOS and p-MOS devices in Supplementary Figure 7, in the case of a As device with $L_{\mathrm{G}} = 7$ nm (similar results are obtained for Sb FETs).

As it can be seen, the n-MOS device shows larger currents and better SS as compared to the p-type device. For this reason, in the main text we decided to focus on n-MOSFETs only, in order to obtain the best performance against Industry requirements.

Supplementary References

[1] Wang, Y. & Ding, Y. Electronic structure and carrier mobilities of arsenene and antimonene nanoribbons: A first-principle study. *Nanoscale Research Letters* **10**, 1–10 (2015).

[2] Bardeen, J. & Shockley, W. Deformation potentials and mobilities in non-polar crystals. *Phys. Rev.* **80**, 72–80 (1950).

[3] Xi, J., Long, M., Tang, L., Wang, D. & Shuai, Z. First-principles prediction of charge mobility in carbon and organic nanomaterials. *Nanoscale* **4**, 4348–4369 (2012).

[4] Herring, C. & Vogt, E. Transport and deformation-potential theory for many-valley semiconductors with anisotropic scattering. *Phys. Rev.* **101**, 944–961 (1956).
